# Acute Appendicitis Complicated by Ascaris lumbricoides Infestation: A Case Report

**DOI:** 10.7759/cureus.97069

**Published:** 2025-11-17

**Authors:** Moataz Mohamed, Mohammed Alshanawani, Abdulelah Alharbi, Fai Algernas, Hazim Abdulrahman

**Affiliations:** 1 General Medicine, The National Ribat University, Khartoum, SDN; 2 General Surgery, King Salman Hospital, Riyadh, SAU

**Keywords:** appendicitis, ascaris lumbricoides, case report, intestinal obstruction, parasitic infestation

## Abstract

Acute appendicitis is one of the most common causes of acute abdomen in surgery. That being said, helminthic infection, namely *Ascaris lumbricoides*, although not a very common cause of appendicitis, when occurring simultaneously, may mimic the same presentation as acute appendicitis, which may prove to be a challenging diagnostic hurdle that may affect the clinical course of patients and their management. We report the case of a 29-year-old female from the Philippines who presented with acute onset of right lower quadrant pain along with vomiting and localized tenderness on examination. Laboratory investigations showed marked leukocytosis. Computed tomography revealed an inflamed appendix along with multiple air-filled linear cavity changes and filling defects seen in the small and large bowel loops. These defects resemble a defect created by worm bodies outlined by intraluminal air and contrast. Laparoscopic appendectomy was uneventful. Postoperative recovery was marked by an episode of vomitus that contained living infestation of *Ascaris*, and the patient was treated with albendazole to eradicate remaining parasites. Histopathological results confirmed acute appendicitis. This case highlights the diagnostic challenge when parasitic infestation coexists with appendicitis, underscoring the importance of considering *Ascaris *in endemic regions.

## Introduction

One of the most prevalent illnesses in the world that causes acute abdominal pain is appendicitis. Appendicitis is primarily caused by lumen blockage. This pathology has been linked to a number of entities. Without a doubt, intestinal ascariasis is among the rarest [1,2]. Globally, over 1.8 billion individuals, or 28% of the world's population, are infected with *Ascaris lumbricoides *because of inadequate hygiene, according to the disease's global distribution and incidence. By 2030, the World Health Organization aims to eradicate at least 75% of pediatric *A. lumbricoides* infection-related morbidity [3]. With that being said, the illness spans a broad spectrum of severity, typically featuring abdominal pain, diarrhea, and vomiting, with the risk of escalating to complications such as intestinal obstruction in severe cases [4]. The primary method for spreading is exposure to the environment through parasite eggs from excrement. Due to this fact, human ascariasis is therefore common in poorer nations where inadequate access to proper water, sanitation, and hygiene resources facilitates transmission from one person to another [5]. With that being said, it takes roughly two to three months to complete the cycle, from ingesting the eggs to producing new ones [6]. In addition to providing a conclusive diagnosis, surgical therapy with appendectomy lowers the risk of complications such as perforation, infection, and death [7]. We describe an instance of laparoscopy being used to treat an accidental diagnosis of appendicitis brought on by an infection. We are presenting the case of a 29-year-old woman who sought treatment for acute pain in the lower right quadrant. Computed tomography (CT scan) with contrast suggested that she had appendicitis and that there were worms in the intestinal lumen.

## Case presentation

In September 2025, a 29-year-old female from the Philippines with no noted comorbidities and no medical or surgical history was admitted to the Department of General Surgery at King Salman Hospital (KSH) in Riyadh, Kingdom of Saudi Arabia.

The patient presented to the Emergency Department with a one-day history of abdominal pain, specifically periumbilical pain, which later shifted to involve the right lower quadrant. She also experienced loss of appetite, nausea, and three previous episodes of vomiting, each containing clear fluid, as described by the patient. One of these episodes was accompanied by nasal regurgitation. The patient did not report any recent travel outside the Kingdom of Saudi Arabia.

Physical examination revealed a patient with normal to stable vitals and a blood pressure of 111/70 mmHg and a heart rate of 87 beats per minute. The abdomen was soft and lax, with moderate abdominal tenderness localized to the right lower quadrant, along with positive rebound tenderness over McBurney’s point. Laboratory workup revealed a white blood cell (WBC) count of 17.00 × 10^9^/L with 84.2% neutrophils, a hemoglobin level of 14.3 g/dL, and a platelet level of 270 x 10^9^/L. Her liver function tests, renal function tests, and urine analysis were unremarkable aside from a potassium level of 3.1 mmol/L, as shown in Table 1.

**Table 1:** Patient's laboratory values on presentation

Test	Patient Value	Normal Range	Unit of Measurement
White blood cells	17.00	3.9-10.2	10^9^/L
Hemoglobin	14.3	12.0-15.6	10^12^/L
Platelets	270	150-375	10^9^/L
Neutrophils %	84.2	42-77	%
Lymphocytes %	9.7	20-44	%
K Level	3.1	3.82-5.49	Mmol/L

**Figure 1:**
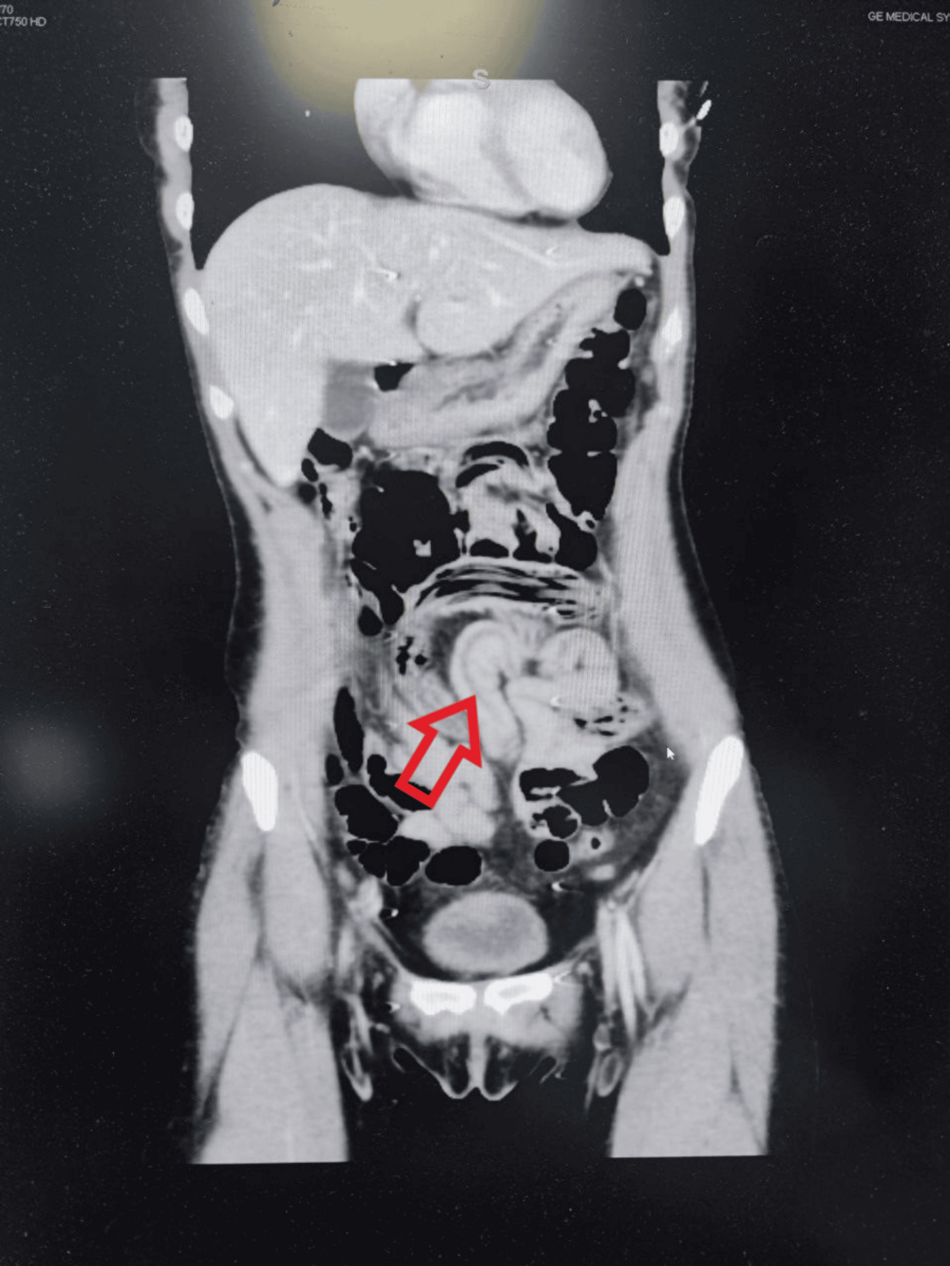
Abdominal computed tomography with intravenous contrast imaging showing air filled linear cavity changes seen in the small and large bowel loops.

Contrast-enhanced CT scan of the abdomen and pelvis revealed an appendix that was medial to the cecum and ascending colon, extending upward anterior to the lower pole of the right kidney. The appendix had a transverse diameter of 0.9 cm with an associated thickened, enhanced wall and peri-appendiceal fat stranding, with no collections observed. Note was made of air-filled linear cavity changes seen in the small and large bowel loops, while the tubular structures were not air-filled. These appearances are highly suggestive of intestinal ascariasis with an air-filled intestinal lumen of the worms. The liver appeared normal, with homogeneous, normal enhancement, and no evidence of focal parenchymal lesions or biliary radicle dilatation. An incidental finding of a left ovarian follicular cyst, as well as a partial filling defect in the right gonadal vein, was also noted. This defect may be related to flow-related changes; however, the possibility of partial thrombosis cannot be excluded. Pelvic vascular congestion was also seen (Figure 1).

**Figure 2:**
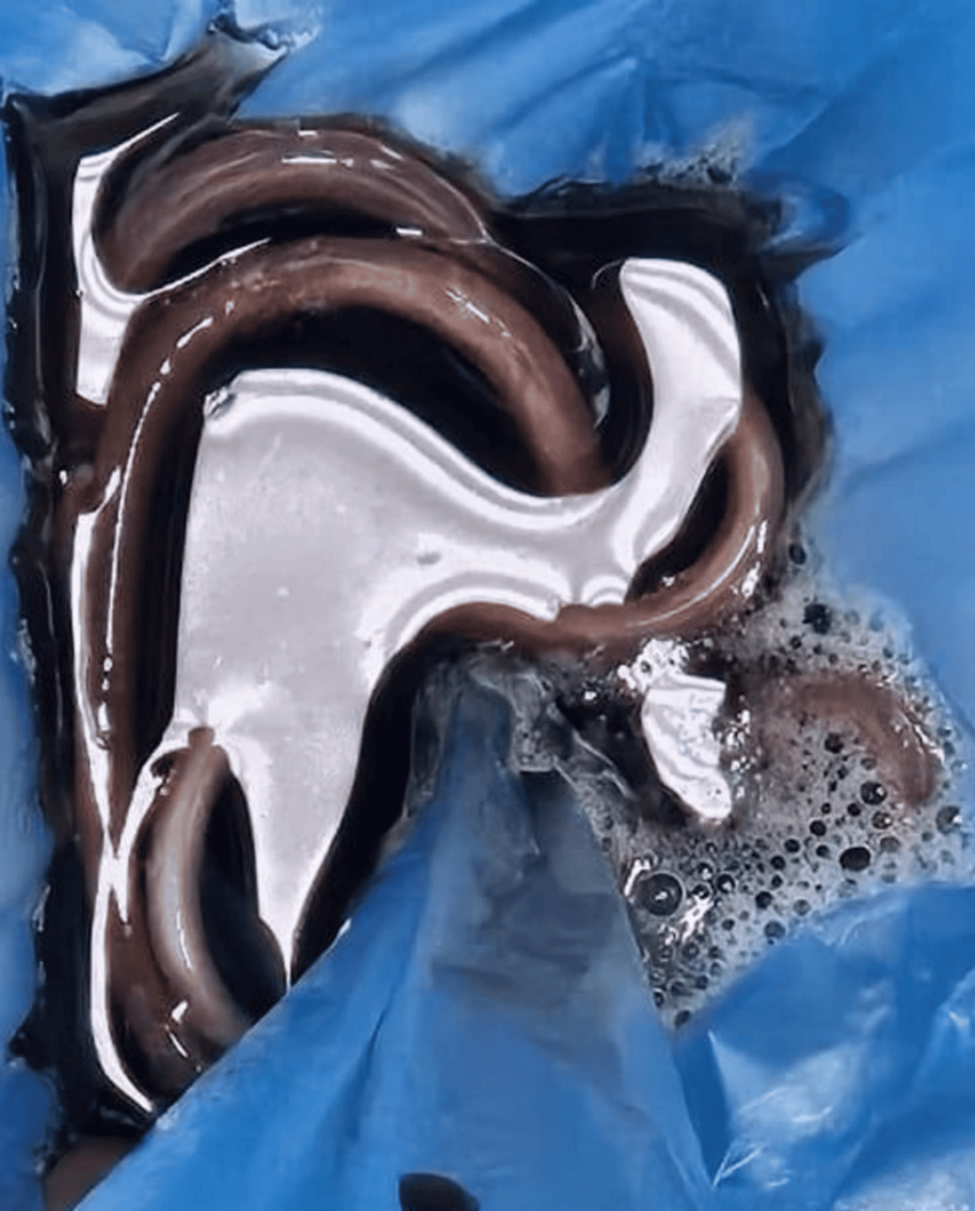
Imaging concerning vomitus illustrating the worms' expulsion.

Following workup, the patient underwent an uneventful laparoscopic appendectomy. The laparoscopic intervention revealed a retrocecal, mildly inflamed appendix, along with free fluid in the abdomen. The right colon was mobilized from the avascular plane using blunt and sharp dissection, after which the appendix was ligated and transected. On the first postoperative day following the laparoscopic appendectomy, the patient experienced an episode of clear liquid vomitus that contained live *Ascaris *worms that were identified on inspection (Figure 2). After close monitoring and multidisciplinary coordination with the Infectious Diseases and Internal Medicine teams, the patient was treated with albendazole, resulting in clinical improvement following combined surgical and medical management.

## Discussion

Epidemiology and parasitic prevalence

With a global age-standardized incidence rate of roughly 214 cases per 100,000 people, or over 17 million new cases annually, appendicitis is a common surgical emergency. The prevalence is lowest in western sub-Saharan Africa (as low as 81.4 per 100,000) and highest in high-income Asia Pacific countries (up to 364 per 100,000) [8]. It is worth mentioning that the most prevalent age range for appendicitis is 10-20 years old, and the male-to-female ratio is 1.4:1. In the US, the lifetime risk is 6.7% for women and 8.6% for men [9].

The human-associated roundworm *A. lumbricoides *is one of the most frequent parasites in the world. Over 1.8 billion individuals worldwide are infected with *A. lumbricoides*, which represents approximately 27% of the world's population [3]. The bulk of ascariasis infections occurs in subtropical and tropical regions of East Asia, the Americas, and sub-Saharan Africa, especially in underprivileged places with inadequate access to clean water and unsanitary circumstances. It is estimated that 13.6% of people in sub-Saharan Africa, 15.6% of people in South America, and 18% of people in Southeast Asia and South Asia are infected with *A. lumbricoides *[10]. A predicted 1.9-5.2 million disability-adjusted life years (DALYs) are caused by *A. lumbricoides *infections worldwide each year. This comprises the years of life lost to early mortality, as well as the years spent disabled by infection-related illness, such as anemia, malnutrition, and delayed cognitive development, particularly in children [11].

Pathophysiology

The stages of ingestion and larval migration are the first to be implicated in the pathophysiology of *A. lumbricoides.* *Ascaris *infections are contracted by hosts through the fecal-oral pathway. It is well known that *Ascaris *larvae develop in a host's parenteral tissues once infectious embryonated eggs are consumed and hatch, releasing the larvae in the small intestine. After that, the larvae break through the intestinal wall and enter the circulation, where they travel to the lungs and alveoli on days six to eight before returning to the respiratory tract and being re-ingested [12]. Once swallowed, the larvae return to the small intestine on approximately days 8-10, where they reside and reach maturation and sexual maturity [13]. The larval migration journey of *Ascaris *triggers various inflammatory responses by the system, notably eosinophilia, and several destructive manifestations on tissue, including the lung, causing different manifestations, such as coughing and wheezing, for which Löffler syndrome is noted [14].

Clinical presentations and diagnostic challenges

Acute appendicitis is primarily diagnosed clinically, and it can show either typical or unusual findings. A lack of appetite, nausea, or vomiting is often present together with undefined peri-umbilical pain that lasts for several hours before moving to the right iliac fossa (RIF). This usual pattern is absent from atypical histories, and discomfort in the lower right quadrant may be the first symptom [15].

According to a study that was conducted in a tertiary care hospital in India, which included 50 patients that underwent emergency appendectomy, 30-50% of appendicitis presentations, depending on the severity of the presentation and the examiner's experience, presented with left iliac fossa deep palpation elicits pain in the RIF, which is termed Rovsing’s sign [16]. Clinical evaluation, physical examination, and laboratory testing (such as an increased white blood cell count) are used to make the diagnosis. Because of its excellent sensitivity and safety, ultrasound is the preferred imaging modality, particularly for young patients. In order to precisely identify low-risk patients, reduce the necessity for imaging, and lower the negative appendectomy rate in these patients, CT scans and scoring systems such as Alvarado and Raja Isteri Pengiran Anak Saleha Appendicitis (RIPASA) are sensitive enough to rule out appendicitis. The use of imaging in patients with a high risk of appendicitis is controversial. A negative imaging scan cannot rule out appendicitis because the condition is very common in this patient population [17].

Management strategies

With regards to the treatment, the standard treatment remains surgical removal of the appendix (appendectomy), either by open or laparoscopic techniques [18]. Anti-helminthic therapy is used to treat illness and its related consequences. Currently, mebendazole (500 mg orally as a single dosage or 100 mg orally twice daily for three days) or albendazole (400 mg orally as a single dose) are the mainstays of treatment for ascariasis [19-21].

## Conclusions

In conclusion, this case serves to highlight that the diagnostic complexity of acute abdomen overlaps with helminthic infection. Acute appendicitis caused by parasite infection is an uncommon occurrence; there is ongoing debate on the connection between parasite infection and appendicitis. The link between acute appendicitis and parasites is not well supported by the available data; however, acute appendicitis can occur when an adult parasite blocks the lumen, but it can also happen when the egg causes a secondary infection. Lymphoid hyperplasia, which results from a secondary infection, is the mechanism causing this condition.

In our case, the reason for acute appendicitis was a secondary infection. We believe that parasitic infections, which may lead to acute abdomen, such as intestinal obstruction and liver abscess, need to be identified via community screening measures before resulting in consequent morbidity and mortality.
